# A Rare Case of Pyoderma Gangrenosum Pointing to Waldenström Macroglobulinemia

**DOI:** 10.1155/crom/5443156

**Published:** 2025-12-26

**Authors:** Laya Krishnan, Emily Vachon, Anvita Mishra, Dawn McCoy

**Affiliations:** ^1^ Carle Illinois College of Medicine, Urbana, Illinois, USA; ^2^ University of Illinois Urbana-Champaign, Champaign, Illinois, USA, illinois.edu; ^3^ Carle Foundation Hospital, Urbana, Illinois, USA, carle.org

## Abstract

**Background:**

Pyoderma gangrenosum (PG) is a rare, noninfectious, truly nongangrenous, autoinflammatory condition marked by neutrophilic dermatosis. It is characterized by the rapid onset of painful, full‐thickness, ulcerative skin lesions with distinctive violaceous and undermined borders. PG is commonly associated with autoimmune and hematologic disorders, namely, inflammatory bowel disease (IBD) and monoclonal gammopathy of undetermined significance (MGUS). However, it has less commonly been reported in association with lymphoplasmacytic lymphoma (LPL) and rarely with its subtype, Waldenström macroglobulinemia (WM).

**Case Presentation:**

This case unfolds the story of a 72‐year‐old female patient with a complex medical and primarily cutaneous oncological history, who initially developed painful lesions on her shins suspected to be PG with a superimposed infection. During extensive infectious, rheumatologic, and oncologic workup revealing an IgM monoclonal gammopathy and antibiotic‐resistant infections, her condition quickly deteriorated with altered mental status and eventual cardiopulmonary arrest 2 months after the initial PG diagnosis.

**Conclusion:**

This case highlights the importance of close follow‐up after PG identification for unusual underlying malignancies and suggests that even an indolent malignancy like WM can contribute to aggressive clinical decline in this setting.

## 1. Introduction

Pyoderma gangrenosum (PG) is an autoinflammatory skin disorder traditionally indicated by painful skin ulcers with violaceous borders on the lower limbs, though lesions can be observed on any part of the body [1]. Pathologically, it is a neutrophilic dermatosis characterized by dense neutrophilic infiltrates within affected tissues; these infiltrates form papules and pustules, which quickly evolve into ulcers [2]. The classical undermined edges of PG ulcers are due to neutrophil‐mediated compromise of the vascular supply and consequent lysis of the dermis, creating a characteristic pocket‐like or overhanging appearance of the ulcer [1, 3]. Additional erythema, edema, and inflammation contribute to PG′s distinctive clinical presentation.

The molecular pathophysiology of PG likely involves innate and adaptive immunity dysregulation. Studies have found elevated levels of proinflammatory cytokines, such as TNF‐*α*, IL‐17, and IL‐23 [4]. Additionally, PG is characterized by pathergy, where minor skin trauma or surgeries can trigger the formation of lesions, likely due to an exaggerated inflammatory response [3]. However, this can often lead to misdiagnosis, since postoperative PG may be mistaken for wound infection or dehiscence [1]. In the postsurgical setting, PG has most often been reported in the context of breast, cardiothoracic, abdominal, and obstetric surgery [5]. Recent literature has identified recurring patterns with symptom presentation (such as a poor response to antibiotics) that may help to raise clinical suspicion in these cases [6].

PG occurs most frequently in tandem with systemic diseases, particularly with autoimmune and hematologic conditions. It has been found to be commonly associated with inflammatory bowel disease (IBD), including ulcerative colitis and Crohn′s disease; studies estimate that nearly 17.6% of PG patients have a concurrent IBD diagnosis [7]. Similarly, studies have shown that hematologic disorders correspond to 9% of PG cases [7]. These include myelodysplastic syndrome, acute myeloid leukemia, and monoclonal gammopathy of undetermined significance (MGUS). Other associations include inflammatory arthritis and solid organ malignancies [7].

While PG has been reported with other hematological malignancies, it has rarely been reported with lymphoplasmacytic lymphoma (LPL) or its subtype, Waldenström macroglobulinemia (WM) [8]. WM is a B‐cell lymphoproliferative disorder characterized by overproduction of IgM and bone marrow infiltrates of lymphoplasmacytic cells > 10%. The excess IgM can lead to hyperviscosity syndrome and end‐organ damage, causing an array of symptoms such as headaches, visual disturbances, anemia, thrombocytopenia, hepatosplenomegaly, and lymphadenopathy [9].

As such, we present a rare case of a patient with no prior history of IBD who rapidly developed ulcerative skin lesions characteristic of PG, with additional workup revealing an elevated serum IgM and a bone marrow biopsy consistent with WM.

## 2. Case Presentation

**Figure 1:**
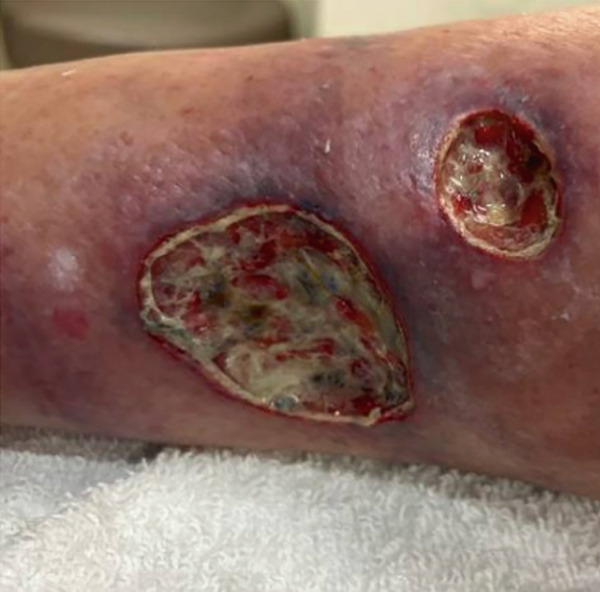
Lesion on right, medial lower leg with measurements 6.7 cm length × 5 cm width × 0.1 cm depth, with an area of 33.5 cm^2^ and a volume of 3.35 cm^3^.

This case highlights a 72‐year‐old female with a 30‐pack‐year smoking history, multiple falls, and a long, complex medical history. Particularly notable conditions include a recent stroke, recurrent pneumonia, chronic hyponatremia, and irritable bowel syndrome. Her oncological history was significant for squamous cell carcinoma on the left forearm 14 years prior, unspecified basal cell carcinoma, and biopsy‐proven mycosis fungoides eight years prior. The case begins with presentation to her primary care physician for evaluation of three painful, friable, serosanguineous, purpuric lesions (the largest measuring 33.5 cm^2^, see Figure 1) across both shins that spontaneously developed a few weeks prior. Dermatologic assessment raised strong suspicion for PG with a superimposed infection. Aerobic cultures of the lesion revealed an overgrowth of *Escherichia coli*, for which she was placed on Bactrim and then cephalexin with little improvement at one week follow‐up. This raised concerns of *Pseudomonas aeruginosa* infection, and she was subsequently placed on levofloxacin with the addition of 100 mg of doxycycline twice daily for seven days for wide‐spectrum coverage while awaiting final culture results. Simultaneously, she was continuing her course of 25 mg of prednisone prescribed for the lesions themselves. The patient would later be admitted to the hospital due to poor healing; however, several tests were conducted in the outpatient setting to explore lesion etiology in the interim, described as follows.

In this same timeframe, the patient′s physicians began exploring rheumatologic or infectious causes for her presentation. Lab testing was negative for RPR; hepatitis A, B, and C; ESR; CRP; and calprotectin (decreasing the likelihood of IBD). Additional negative results included antinuclear antibodies, antineutrophil cytoplasmic antibodies, anti‐centromere antibodies, SCL‐70 antibodies, rheumatoid factor, anticyclic citrullinated peptide, antiphospholipid antibodies, and anti‐beta‐2 glycoprotein 1 antibodies. However, an immunotyping assay found an abnormally elevated SPEP‐IgM of 746.8 mg/dL (normal range 33.0–293.0 mg/dL) with a monoclonal peak of 0.5 g/dL. The assay also demonstrated an increased serum kappa/lambda FLC ratio of 2.83 (normal ratio 0.26–1.65) and elevated levels of both alpha‐1 globulin (0.41 g/dL) and gamma globulin (1.73 g/dL). The patient was referred to hematology for further evaluation. Repeated electrophoresis and immunotyping revealed a further elevated kappa/lambda FLC ratio (3.00) and consistently elevated IgM (649.8 mg/dL) and monoclonal peak (0.38 g/dL). Table S1 provides greater detail with a time course of the patient′s complete blood count values during her last two weeks of life.

One month after antibiotic initiation, the patient was admitted to the hospital due to poor wound healing. Wound cultures found that the lesions were now growing antibiotic‐resistant *Enterococcus faecalis*, *Escherichia coli*, and *Citrobacter freundii*, although blood cultures revealed no growth. She was consequently started on 1 g of intravenous (IV) cefepime every six hours along with IV linezolid, and infectious disease specialists were consulted. They recommended discontinuation of cefepime upon discharge and starting a two‐week outpatient course of both IV ertapenem daily (received through a PICC line) and oral linezolid twice daily. The wound care team was also on board to help manage the lesions, supplementing the lesion treatment with Santyl and compression wraps. Over this time course, there was slight improvement in infection clearance. However, wound appearance remained largely unchanged, further pointing toward a noninfectious underlying cause. A prednisone taper was overseen by a consulted dermatologist, who also agreed to proceed with a biopsy following discharge.

Concurrent oncological workups during her admission included fluorodeoxyglucose‐18 positron emission tomography (F‐18 FDG PET) scans and a bone marrow biopsy to rule out multiple myeloma. The scans demonstrated abnormal radiotracer accumulation in four locations: the esophagogastric junction, gastric antrum, proximal duodenum, and notably, within a small nodule in the right lower lobe of the lung suspicious for malignancy. Bone marrow biopsy of the right iliac crest revealed LPL with involvement of 50%–60% clonal cells, establishing a primary diagnosis. It additionally contained background aspirates showing decreased iron stores (which help to explain her persistent anemia) and marrow showing scattered erythroid precursors with cytoplasmic vacuolization. This marrow presentation is typically associated with VEXAS syndrome, nutritional deficiencies (such as vitamin B12, folate, copper, and zinc), and as a paraneoplastic response, given the coexisting LPL [10].

Two months after initial presentation for her skin lesions, and eight days after hospital discharge, the patient developed a sore throat diagnosed as thrush. She was treated with oral clotrimazole, after which she developed extreme dizziness, sharp abdominal pain, and nausea. Five days after starting clotrimazole, the patient was experiencing altered mental status for which emergency medical services were called. Upon their arrival, the patient was bradycardic, hypotensive, and hypoglycemic (blood glucose < 20 mg/dL). En route to the emergency department (ED), she was started on 10% dextrose (D10W) and subsequently lost her pulse, after which ACLS protocol and CPR were initiated. Despite two administrations of epinephrine, the patient had bloody secretions in the oropharynx on ED arrival and died of cardiopulmonary arrest. Interestingly, her postmortem Epstein–Barr virus (EBV) viral capsid antigen IgG and nuclear antigen antibodies were dramatically elevated at > 750.0 and > 600.0 U/mL, respectively.

## 3. Discussion

PG is often associated with hematologic malignancies and disorders, and this association is even stronger in patients older than 65 [11]. Of the plasma cell dyscrasias, MGUS is most commonly associated with PG. Estimates of MGUS prevalence among patients with PG may be as high as 27% [12]. However, the vast majority of patients with MGUS and PG have an IgA and/or IgG monoclonal gammopathy, with IgA being most common. Similarly, in patients with multiple myeloma and PG, IgA multiple myeloma is most prevalent [8]. Researchers have proposed several mechanisms as to why the IgA subtype of MGUS and multiple myeloma is most strongly correlated with PG. One leading theory proposes that IgA immunoglobulins activate neutrophils via IgA receptors, but then inhibit neutrophil function, thereby affecting the healing of the skin and potentially leading to PG [13].

However, there are very few cases documented in the literature of PG and IgM gammopathy. The patient in this report met the criteria for WM given her IgM spike and bone marrow involvement of 50%–60% clonal lymphoplasmacytic cells and was thus diagnosed clinically. A recent systematic review by Montagnon et al. identified 340 cases of PG with hematological malignancies. Of those cases, only three were correlated with WM. Upon literature search, we were unable to identify any standalone case reports of PG with WM (or IgM MGUS), substantiating this rarity. Furthermore, cutaneous manifestations of IgM gammopathy in general are uncommon but can occur due to paraproteinemia, paraprotein‐related autoimmune diseases, and most rarely, infiltration of the skin by neoplastic cells [14, 15]. In the literature, there are a few cases of ulcerations in WM patients found to be a result of direct IgM deposition in small vessels [16, 17]. Though PG was strongly suspected in this patient, histopathological confirmation through biopsy was never completed due to the patient′s unexpected death. There is a small possibility that her lesions were in fact paraprotein‐related ulcers, or another etiology, rather than PG, an important caveat in this case.

When it comes to treatment, PG patients with monoclonal gammopathy tend to have more frequent recurrence and poorer responses to standard treatment [12]. This is consistent with our patient′s case, as her lesions continued to worsen and provide a nidus for bacterial infections despite intensive steroid and antibiotic treatment. Typically, the underlying gammopathy needs to be treated in order to resolve the skin lesions [18]. This can be a challenging decision when the patient appears otherwise asymptomatic and shows no signs of systemic disease, as was the case with this patient. Retrospectively, this patient may have been experiencing hyperviscosity symptoms which were overlooked due to her complex medical history. She had a history of cardiovascular comorbidities, chronic migraines, and mild to moderate thrombocytopenia, as well as lower extremity neuropathy which was first noted about six months before her PG lesions appeared. She also experienced a sudden rectus sheath hematoma three months prior to the onset of her PG, and her husband reported that the patient developed severe dizziness and ataxia two days before her death. Hyperviscosity may have also added strain to her already tenuous cardiac status, eventually contributing to her demise. Though not clearly definable, the reasons for her rapid decline and death were likely multifactorial. Factors include an uncontrolled autoinflammatory skin process (PG), hyperviscosity, and immunocompromise as a result of malignancy and immunosuppressive therapy, ultimately leading to septic shock (given her abnormal vital signs).

A final consideration is that while we believe that the patient′s WM was associated with her PG, it is also possible that she had another contributing malignancy. Her F‐18 FDG PET scan demonstrated abnormal radiotracer uptake in the lung and multiple locations along the gastrointestinal tract. Solid organ malignancies are associated with WM at about the same rate as hematologic malignant neoplasms [11]. However, the patient′s death precluded any follow‐up scans, biopsies, or genetic testing for the MYD88 L265P mutation prevalent in around 90% of WM cases [19]. In a similar line, the patient′s elevated levels of EBV viral capsid antigen IgG and nuclear antigen antibodies are a curious finding. Whether this observation was purely incidental, an effect of the patient′s immunosuppressive state, or if reactivation may have contributed to the patient′s deterioration through an occult infection or by triggering a hemophagocytic process, it remains another factor for further exploration, had it been feasible.

## 4. Conclusion

Although it is not uncommon for hematologic disorders to underlie a PG diagnosis, this case highlights the importance of timely and proactive oncological work‐up, even when the patient is not experiencing red flag symptoms. Additionally, the case demonstrates a very rare association between WM and PG, encouraging clinicians not to underestimate this avenue in the assessment of patients with suspicious cutaneous manifestations and clinical deterioration. An underlying monoclonal gammopathy, even if asymptomatic, can significantly hinder PG healing, and the presence of atypical features may point toward subtle hyperviscosity syndrome. Dermatologists and oncologists may find these insights particularly useful.

## Consent

Written informed consent was obtained from the patient′s family for publication of this case report.

## Conflicts of Interest

The authors declare no conflicts of interest.

## Funding

No funding was received for this manuscript.

## Supporting information


**Supporting Information** Additional supporting information can be found online in the Supporting Information section. Attached as supporting information is a table of trending complete blood counts over the patient′s last 2 weeks of life.

## Data Availability

Data sharing not applicable to this article as no datasets were generated or analysed during the current study.
