# Supplementary material for: A Rare Case of Pyoderma Gangrenosum Pointing to Waldenström Macroglobulinemia
Source: Case Rep Oncol Med. 2025 Dec 26;2025:5443156. doi: 10.1155/crom/5443156 (PMC12747114; doi:10.1155/crom/5443156)
Supplement: Supplementary file 1 — Supporting Information Additional supporting information can be found online in the Supporting Information section. Attached as supporting information is a table of trending complete blood counts over the patient′s last 2 weeks of life. [file CROM-2025-5443156-s001.docx]

| **Tab.S1:** Trends of CBC over the patient’s last two weeks of life. Absolute neutrophil counts are consistently elevated. Values in blue are lower than given range; values in red are higher than given range. | | | | | | | | |
| --- | --- | --- | --- | --- | --- | --- | --- | --- |
| **Value** | **Range** | **Four days before** | **Seven days before** | **Thirteen days before** | **Sixteen days before** | **Seventeen days before** | **Eighteen days before** | **Nineteen days before** |
| WBC | 4.00 - 11.00 10^3/uL | 9.87 | 11.41 | 9.81 | 9.24 | 10.30 | 11.91 | 10.34 |
| RBC | 3.50 - 5.20 10^6/uL | 2.65 | 3.09 | 3.00 | 2.98 | 3.10 | 3.52 | 3.45 |
| HGB | 11.0 - 16.0 g/dL | 8.8 | 10.2 | 9.9 | 9.7 | 10.1 | 11.4 | 11.3 |
| HCT | 34.0 - 47.0 % | 25.9 | 30.8 | 29.8 | 29.8 | 30.9 | 34.8 | 34.1 |
| MCV | 80.0 - 100.0 fL | 97.7 | 99.7 | 99.3 | 100.0 | 99.7 | 98.9 | 98.8 |
| MCH | 26.0 - 33.0 pg | 33.2 | 33.0 | 33.0 | 32.6 | 32.6 | 32.4 | 32.8 |
| MCHC | 31.0 - 35.0 g/dL | 34.0 | 33.1 | 33.2 | 32.6 | 32.7 | 32.8 | 33.1 |
| RDW | 12.0 - 15.0 % | 13.5 | 13.9 | 14.4 | 13.9 | 13.8 | 13.9 | 13.7 |
| RDW-SD | 38.0 - 52.0 fL | 48.6 | 51.2 | 52.1 | 51.5 | 50.7 | 50.4 | 49.8 |
| PLATELET | 140 - 400 10^3/uL | 50 | 104 | 165 | 145 | 164 | 200 | 199 |
| MPV | 9.0 - 12.0 fL | 10.5 | 9.3 | 9.5 | 9.4 | 9.4 | 9.3 | 9.4 |
| SEG | % | 88.1 | 87.2 | 83.7 | 81.3 | 78.1 | 92.7 | 91.7 |
| LYMPHOCYTE | % | 7.6 | 7.2 | 8.0 | 9.0 | 7.9 | 3.5 | 3.9 |
| MONOCYTE | % | 3.0 | 3.6 | 6.8 | 7.3 | 10.9 | 2.5 | 3.0 |
| EOSINOPHIL | % | 0.5 | 1.0 | 0.6 | 1.6 | 1.7 | 0.1 | 0.2 |
| BASOPHIL | % | 0.2 | 0.3 | 0.3 | 0.3 | 0.3 | 0.2 | 0.1 |
| IMMATURE GRANULOCYTE | % | 0.6 | 0.7 | 0.6 | 0.5 | 1.1 | 1.0 | 1.1 |
| ABSOLUTE NEUTR | 1.60 - 7.70 10^3/uL | 8.69 | 9.96 | 8.21 | 7.51 | 8.06 | 11.04 | 9.49 |
| ABSOLUTE LYMPH | 1.00 - 4.90 10^3/uL | 0.75 | 0.82 | 0.78 | 0.83 | 0.81 | 0.42 | 0.40 |
| ABSOLUTE MONO | 0.00 - 1.10 10^3/uL | 0.30 | 0.41 | 0.67 | 0.67 | 1.12 | 0.30 | 0.31 |
| ABSOLUTE EOS | 0.00 - 0.50 10^3/uL | 0.05 | 0.11 | 0.06 | 0.15 | 0.17 | 0.01 | 0.02 |
| ABSOLUTE BASO | 0.01 - 0.20 10^3/uL | 0.02 | 0.03 | 0.03 | 0.03 | 0.03 | 0.02 | 0.01 |
| ABSOLUTE IMMATURE GRANULOCYTE | 0.00 - 0.09 10^3/uL | 0.06 | 0.08 | 0.06 | 0.05 | 0.11 | 0.12 | 0.11 |
